# The Association Between Early Progesterone Rise and Serum Estradiol Levels as Well as Endometrial Thickness in IVF Cycles

**DOI:** 10.3390/jcm14175965

**Published:** 2025-08-23

**Authors:** Katarina Ivanovic, Lidija Tulic, Ivan Tulic, Stefan Ivanovic, Jelena Stojnic, Jovan Bila, Tatjana Dosev, Zeljka Vukovic, Branislav Milosevic

**Affiliations:** 1 Clinic for Gynecology and Obstetrics, University Clinical Center of Serbia, 11000 Belgrade, Serbia; ikatarina.1996@gmail.com (K.I.); tulicivan@yahoo.com (I.T.); ivfjelena@gmail.com (J.S.); bilamsj@gmail.com (J.B.); dosevt@gmail.com (T.D.); drwishies@gmail.com (Z.V.); drbanemilosevic@gmail.com (B.M.); 2 Faculty of Medicine, University of Belgrade, 11000 Belgrade, Serbia; 3 Obstetrics and Gynecology Clinic “Narodni Front”, 11000 Belgrade, Serbia; ivanoovic93@gmail.com

**Keywords:** endometrial thickness, E2 levels, hCG trigger, P4

## Abstract

**Background/Objectives:** The success of artificial reproductive technologies (ARTs) depends on different factors, such as patient-specific reproductive features, ovarian response to stimulation, oocyte and embryo quality, and endometrial receptivity. This study aimed to evaluate their association with oocyte yield, fertilization, endometrial thickness, and pregnancy outcomes. **Methods:** A prospective clinical study included 128 women undergoing IVF/ICSI. Baseline hormone levels (E2, P4, FSH, LH, AMH) were assessed prior to stimulation. E2 levels were monitored during stimulation, and P4 was measured on the day of oocyte retrieval. Patients were grouped based on P4 levels (<2 ng/mL vs. ≥2 ng/mL). IVF outcomes and endometrial characteristics were statistically analyzed. **Results:** Lower P4 levels (<2 ng/mL) on the day of oocyte retrieval were significantly associated with higher fertilization rates (*p* < 0.003), more fertilized oocytes (*p* < 0.001), and increased pregnancy rates (*p* < 0.001). Elevated P4 (≥2 ng/mL) correlated with a higher frequency of thin endometrium (<7 mm, *p* < 0.007). E2 levels on the hCG trigger day correlated positively with the number of retrieved and mature oocytes and fertilization outcomes (*p* < 0.05). Patients who achieved pregnancy had lower P4 and BMI, and higher E2, AMH, and endometrial thickness. ROC identified a P4 threshold of 1.99 ng/mL with moderate predictive value. **Conclusions:** Elevated progesterone levels on the day of oocyte retrieval negatively impact fertilization and pregnancy outcomes, likely due to impaired endometrial receptivity. Combined assessment of P4, E2, AMH, and endometrial thickness may enhance embryo transfer planning and improve IVF success rates.

## 1. Introduction

The success of artificial reproductive technologies (ARTs) depends on different factors, such as patient-specific reproductive features, ovarian response to stimulation, oocyte and embryo quality, and endometrial receptivity. Basal hormonal status and hormonal status during controlled ovarian stimulation have a significant role in assessing prognostic parameters that influence the outcomes [1]. Among the most frequently evaluated endocrine markers are estradiol (E2) and progesterone (P4), whose serum levels at specific phases of the cycle—e.g., the levels of estradiol (E2) on the day of hCG administration and progesterone (P4) on the day of oocyte retrieval—may significantly impact embryo development, implantation potential, and clinical pregnancy achievement [2,3]. Serum estradiol concentrations during the early follicular phase, as well as throughout the stimulation cycle, are considered indirect indicators of follicular function and potential predictive markers of ovarian response. However, the interpretation of both basal and stimulated E2 levels remains controversial. While elevated early follicular E2 may reflect advanced follicular recruitment, it can also mask elevated FSH values and potentially indicate a diminished ovarian reserve and suboptimal response later in the cycle [4,5]. Moreover, estradiol is viewed as a potential marker of endometrial receptivity. Its dynamic increase during stimulation significantly influences endometrial proliferation and thickness, parameters which are often used to guide embryo transfer decisions. Clinically, studies showed that an endometrial thickness of 7 mm or more on the day of hCG administration is generally considered favorable for implantation, whereas a thinner endometrium has been associated with significantly lower pregnancy rates. Nevertheless, excessively high E2 concentrations, particularly in high responders, may lead to premature endometrial maturation and histologic asynchrony relative to embryo development, thereby reducing implantation potential even when endometrial thickness appears adequate. Thus, while endometrial thickness may correlate with E2 levels, it is not a reliable marker of functional receptivity [6,7]. Additionally, progesterone does not directly affect endometrial thickness but is essential for secretory transformation, synchronizing endometrial and embryonic development, and establishing the implantation window. Premature progesterone rise may induce histologically advanced but functionally unreceptive endometrium. Therefore, clinical practice often recommends freezing all embryos in such cycles and postponing transfer to a subsequent, hormonally optimized cycle [8].

Indeed, while prior studies have explored the individual effects of estradiol or progesterone on IVF outcomes, there is a notable lack of studies that simultaneously investigate the interrelationship between serum progesterone levels at oocyte retrieval, estradiol concentrations, and endometrial thickness within the same cycle. Our study aims to address this gap by evaluating whether a concurrent assessment of these parameters can better predict clinical pregnancy rates, thus offering potentially useful insights for individualized clinical decision-making in fresh transfer IVF cycles [9]. Given these considerations, the aim of this study was to evaluate the association between serum estradiol during stimulation and progesterone on the day of oocyte retrieval with IVF outcomes, including the potential application of ‘freeze-all’ protocols in cycles with elevated progesterone.

Greater emphasis was placed on the predictive value of hormonal levels for oocyte yield, fertilization, pregnancy success, and endometrial thickness. Establishing optimal hormonal thresholds could enable more precise cycle monitoring, tailored treatment, and better outcomes.

## 2. Materials and Methods

A retrospective clinical study was conducted at the Clinic for Gynecology and Obstetrics, University Clinical Center of Serbia. The study included 128 female patients undergoing in vitro fertilization (IVF) treatment at the Clinic. All patients were less than 44 years old, their body mass index (BMI) was less than 30 kg/m^2^, and infertility was caused by male factors, ovarian dysfunction, tubal factors, combined causes, or unknown factors. All embryo transfers were performed in fresh cycles on day two or three, without prior embryo cryopreservation. All participants received information about the purpose of the study, and they provided written consent to participate. The study was approved by the Ethics Committee of the Faculty of Medicine, University of Belgrade.

Inclusion criteria encompassed women aged 18 to 40 years with a body mass index (BMI) between 18 and 30 kg/m^2^, regular menstrual cycles (ranging from 25 to 32 days), and no major medical conditions or advanced endometriosis (stage III or IV). For all participants, data were collected on age, BMI, duration of infertility, smoking status (smoker or non-smoker), and the underlying cause of infertility. The etiology of infertility was classified as male factor, tubal, ovarian, unexplained, or combined. Stimulation protocols were selected on an individual basis, taking into account patient age, ovarian reserve, and previous IVF outcomes. Accordingly, patients underwent either a short GnRH antagonist protocol (*n* = 112) or a long GnRH agonist protocol (*n* = 16). On the second or third day of the menstrual cycle, before beginning IVF treatment, all patients underwent baseline hormonal testing and ultrasound examination. Serum levels of estradiol (E2), follicle-stimulating hormone (FSH), luteinizing hormone (LH), and anti-Müllerian hormone (AMH) were measured. Patients on the long stimulation protocol with GnRH agonists received Triptorelin 0.1 mg (Diphereline, Ipsen Pharma Biotech, Paris, France) from the mid-luteal phase of the previous cycle, and from cycle day 2 or 3, ovarian stimulation with recombinant FSH (Gonal-F, Serono, Geneva, Switzerland) was started. Patients on the protocol with GnRH antagonists began the same gonadotropin stimulation on the second or third day of the cycle, with the GnRH antagonist cetrorelix (Cetrotide, Merck Serono, Darmstadt, Germany) introduced on the 6th day of stimulation. In both ovarian stimulation protocols, the ovarian response was monitored through sequential transvaginal ultrasound examinations and serum estradiol measurements- on day 6, day 8, and the day of hCG trigger. Human chorionic gonadotropin (Pregnyl, Organon, Oss, The Netherlands) was administered at a dose of 5000 IU, 34–36 h before oocyte retrieval. The retrieved oocytes were classified based on their maturation stage as either mature (MII) or immature (MI). Fertilization was confirmed 16–20 h post insemination. The fertilization rate refers to the proportion of fertilized MII oocytes. Embryo quality was assessed according to the Istanbul Consensus criteria [10,11]. Embryo transfer was performed on day two or three after oocyte retrieval, with a maximum of three embryos transferred. Luteal support started on the day of oocyte retrieval with intramuscular progesterone depot injections. Pregnancy was confirmed 14 days after embryo transfer.

Serum levels of E2—pg/mL, P4—ng/mL, FSH—mIU/mL, and LH—mIU/mL, AMH (ng/mL) were measured on the day two or three of the menstrual cycle—baseline hormonal profile. During stimulation, estradiol levels were monitored on days 6, 8, and on the day of hCG trigger, and progesterone levels were additionally measured on the day of oocyte retrieval. Blood samples were taken by Vacutainer tubes (BD Vacutainer Systems) and centrifuged; the measurement methods are explained in a previous study [12]. Briefly, the AMH value in serum was measured by ELISA (enzyme-linked immunosorbent assay), and other hormones, namely FSH, LH, E2, and P4, were analyzed by chemiluminescent immunoassay.

### 2.1. Embryo Transfer Protocol

Embryo transfers were performed on day 2 or day 3 post oocyte retrieval, according to the clinical practice at our center. All embryos were cultured in sequential media under standard conditions, and selection for transfer was based on morphological criteria available at the cleavage stage. Luteal phase support was initiated on the day of oocyte retrieval and continued until the pregnancy test and beyond if pregnancy was achieved.

### 2.2. Statistical Analysis

For statistical analysis of the obtained data, we applied methods of descriptive and analytical statistics. Descriptive statistics were used to summarize the characteristics of the analyzed patients, including basic demographic data, hormonal profiles, clinical parameters, and individual and combined risk factors in relation to progesterone (P4) levels on the day of oocyte retrieval (OR). Data for continuous variables are presented as mean values and standard deviations (SD), while for discontinuous ones, frequencies were determined and are shown in the form of patient number and percentage of total population. Differences between examined parameters regarding the levels of P4 and E2 were assessed regarding the exact level using ANOVA as well as concerning the P4 and E2 level groups (low, intermediate and high) applying Kruskal–Wallis χ^2^ (non parametric ANOVA). Receiver Operator Curve analysis (ROC) was used in order to determine the optimal range of cut-off levels for P4 at OR and E2 at hCG trigger time for achieving pregnancy and to evaluate the reliability of P4 and E2 as predictors of successful pregnancy realization. The associations between the parameters and obtained oocytes and embryos, as well as pregnancy numbers, from examined patients were evaluated by Spearman correlations (ρ). Finally, the Enter method of logistic regression was applied in order to investigate possible predictors of obtained and mature oocytes and fertilization rate. The level of significance was *p* < 0.05. Obtained data were analyzed using SPSS 22.0 software (SPSS Inc., Chicago, IL, USA).

## 3. Results

The study included 128 women with a mean age of 34.54. The average BMI of the investigated women was 22.39. From the total number of women, the mean level of P4 on the day of OR was 2.28 ng/mL and E2 on the day of hCG was 2330.21 pg/mL. (Table 1). In Table 2, the baseline characteristics of patients and procedure outcome depending on P4 level on the day of OR are presented. There were significantly more women who had a BMI < 25, (χ^2^ = 52.53; *p* = 0.001). Most patents had endometrium thickness > 7 mm (*n* = 116; 90.63%), but the endometrium was thinner (<7 mm) in those patients who had higher P4 levels (>2), *p* < 0.007. The majority of patients did not smoke (χ^2^ = 34.03; *p* = 0.001). The most frequent infertility causes were male (*n* = 34; 26.6%) and unknown factor (*n* = 35; 26.4%). They have been treated for infertility from 1,5 to 13 years. Antagonist protocol was applied in the majority of women (χ^2^ = 72,00; *p* = 0.001). There were no significant differences regarding the insemination method (χ^2^ = 1.504; *p* = 0.471). There were no significant differences between women who have achieved pregnancy and those who did not (χ^2^ = 0.281; *p* = 0.596). There were significant differences between women that have achieved pregnancy and a lower level of P4 and those who had a higher level of P4 (*p* < 0.001). In the group of women who obtained pregnancy, the mean level of P4 on the day of OR was 1.95 ng/mL, and E2 on the day of hCG was 2622.70 pg/mL, while for those who were not pregnant the average P4 was 2.64 ng/mL, and E2 was 2008.95 pg/mL. Differences between examined parameters (mean values) regarding the level of progesterone on the day of OR are presented in Table 3. There were no significant differences between the examined parameters regarding higher and lower levels of progesterone. However, if progesterone levels were evaluated in range groups, significant differences were registered for levels of E2 at the time of hCG trigger as well as the fertilization rate. Correlations between examined patients’ characteristics and progesterone on the day of OR, estradiol on the day of hCG trigger, mature oocyte number, and pregnancy are presented in Table 4. The patient’s age was correlated with the level of E2 on hCG day, the number of MII oocytes and endometrium thickness. The level of FSH was negatively correlated with the level of estradiol on hCG trigger and MII oocytes. The level of AMH was also correlated with the level of E2 on hCG day, MII oocytes, and endometrium thickness. The level of estradiol on the trigger day was correlated with the level of progesterone on the day of OR, with M II oocytes and pregnancy. In Table 5, we can see that the number of obtained oocytes as well as the number of mature (MII) oocytes were correlated with the patient’s age, with the level of estradiol on trigger day, and endometrium thickness. Fertilization rate was negatively correlated with the level of progesterone on the day of OR, E2 on the day of hCG, and with pregnancy. The number of achieved pregnancies was negatively correlated with progesterone levels assessed in different ranges. Table 6 shows that patients with a positive outcome of the IVF procedure had statistically lower BMI (*p* < 0.016) and P4 on OR day (*p* < 0.001), while statistically higher AMH (*p* < 0.05), and E2 on hCG day (*p* < 0.023), as well as number of received oocytes (*p* < 0.050), and fertilization rate (*p* < 0.003). Univariate logistic regression analysis suggested that endometrium thickness more than 7 mm, a higher level of estradiol on the day of hCG trigger, as well as a higher number of received oocytes and positive outcome of the procedure present univariate predictors of P4 < 2 level in all IVF cycles. To avoid any misunderstanding, we have revised the manuscript to clarify that our findings indicate a correlation—not causation—between progesterone levels and endometrial thickness. We also emphasize that this association may represent an indirect marker of endometrial receptivity or hormonal environment quality, rather than a direct effect of progesterone on endometrial proliferation. Higher AMH levels and higher fertilization rate are also potential predictors. Multivariate logistic regression analysis showed that levels of P4 lower than 2 are predictors of a positive outcome (Table 7).

**Figure 1:**
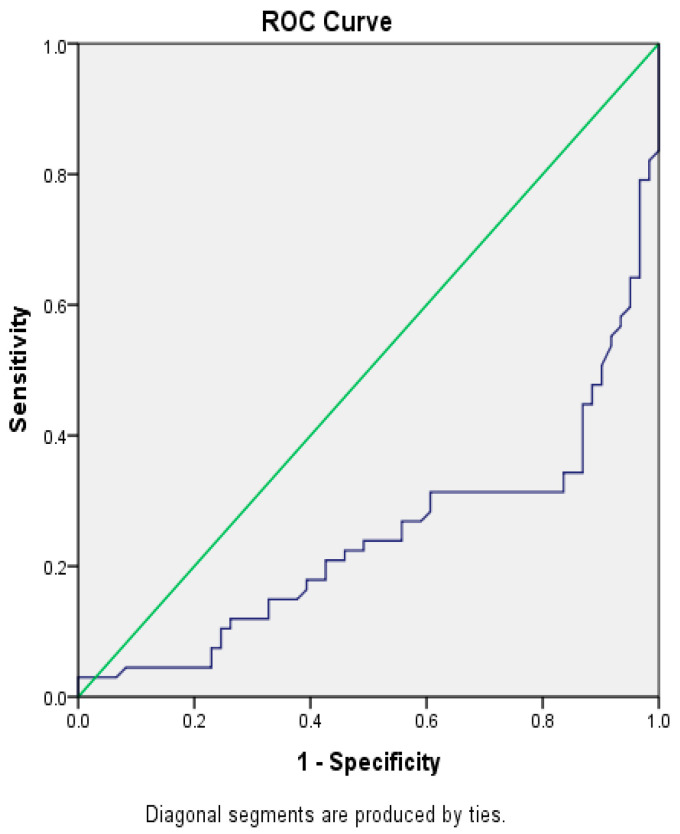
ROC curve for serum progesterone levels on oocyte retrieval day predicting clinical pregnancy. Optimal cut-off: 1.99 ng/mL (AUC = 0.742, *p* < 0.001). The blue line represents the ROC curve, demonstrating the discriminative ability of serum progesterone levels on the day of oocyte retrieval in predicting clinical pregnancy. The green diagonal line represents the line of random prediction, serving as a reference indicator for assessing the model’s accuracy.

**Figure 2:**
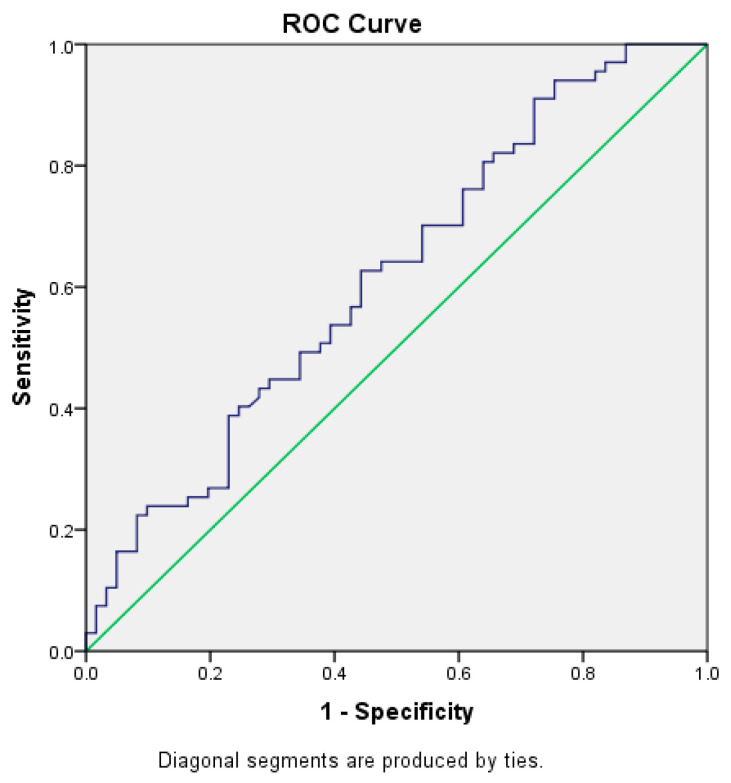
ROC curve for serum estradiol levels on hCG trigger day predicting clinical pregnancy. Optimal cut-off: 857 pg/mL (AUC = 0.617, *p* = 0.046). The blue line represents the ROC curve, illustrating the predictive value of serum estradiol levels on the day of hCG administration for achieving clinical pregnancy. The green diagonal line represents the reference line of random prediction, used for comparison with the discriminative ability of the analyzed parameter.

**Figure 3:**
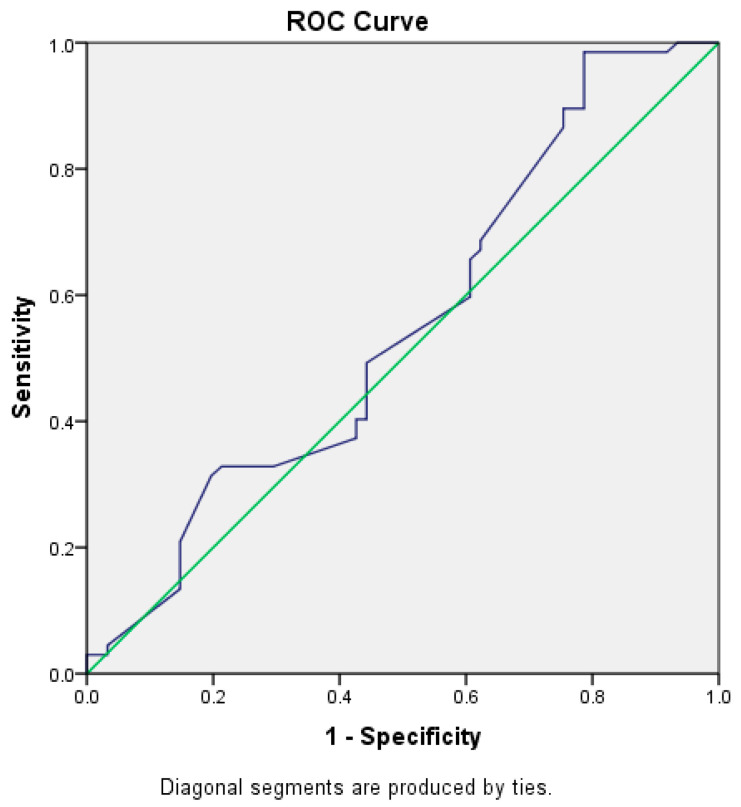
ROC curve for endometrial thickness as a predictor of clinical pregnancy. Optimal cut-off value of endometrial thickness was ≥9 mm, with a sensitivity of 75.8% and specificity of 52.4%. The blue line represents the ROC curve, indicating the ability of endometrial thickness to predict the probability of achieving clinical pregnancy. The green diagonal line represents the line of random prediction, serving as a reference baseline for evaluating the performance of the predictive model.

Based on the ROC, the optimal range of cut-off levels for progesterone on the day of OR in order to achieve pregnancy is 1.99 ng/mL (sensitivity = 68.7%; specificity = 80.3%), Area = 0.242, *p* < 0.000 (Figure 1). The obtained cutoff was in accordance with literature data (11–13). Therefore, the level of P4 on the day of OR was assessed as continuous as well as low (<2 ng/mL) and high/elevated (≥2 ng/mL). The optimal range of cut-off levels for estradiol on the day of hCG trigger in order to achieve pregnancy is 857 pg/mL (sensitivity = 89.6%; specificity = 30%), Area = 0.617, *p* < 0.023 (Figure 2). The optimal range of cut-off levels for endometrium thickness at the end of stimulation in order to achieve pregnancy is 6.9 mm (sensitivity = 98.5%; specificity = 10.1%) Area = 0.551, *p* = ns (Figure 3).

## 4. Discussion

The overall clinical pregnancy rate in our cohort was 52.3%. This relatively high success rate can be attributed to the favorable patient characteristics in our study population, including younger age (mean age: 34.5 ± 4.1 years), good ovarian reserve (mean AMH: 2.72 ± 2.70 ng/mL), and a relatively high number of retrieved oocytes (mean: 8.93 ± 7.06). Additionally, the study included mostly patients undergoing their first or second IVF cycle. The results of our study showed that baseline hormonal values, as well as serum levels of estradiol (E2) and progesterone (P4) during controlled ovarian stimulation, significantly influence IVF outcomes. In our cohort, progesterone concentrations on the day of oocyte retrieval below 2 ng/mL were linked with higher fertilization and pregnancy rates. These findings are consistent with other studies identifying elevated P4 levels in the late follicular phase as a potentially detrimental factor for implantation, likely due to premature closure of the implantation window caused by early secretory transformation of the endometrium [13,14,15]. The adverse impact of elevated P4 on ART outcomes appears to be primarily endometrial rather than oocyte-related, which is supported by our findings. Several studies have shown that even with favorable embryological parameters, high P4 may induce histological asynchrony between the endometrium and embryo [16,17]. In our analysis, patients with P4 ≥ 2 ng/mL had a significantly higher proportion of endometria thinner than 7 mm, potentially indicating impaired proliferative and secretory transformation. Although endometrial thickness itself did not reach statistical significance as a predictor of pregnancy in the ROC, it remains an important clinical parameter, particularly when interpreted in conjunction with hormonal profiles.

Serum estradiol (E2) levels on the day of hCG administration were positively and significantly correlated with the total number of retrieved and mature oocytes, fertilized oocytes, fertilization rate, and pregnancy achievement. This supports the role of E2 as a reliable marker of follicular development and functional ovarian reserve. However, some studies suggest that excessively elevated E2 levels, typically observed in high responders, may compromise endometrial receptivity and reduce implantation rates, although our data did not directly confirm this [18]. Beyond the individual effects of progesterone and estradiol, our results highlight the importance of their interaction. Among patients with P4 < 2 ng/mL, significantly higher E2 levels on the day of hCG administration and greater endometrial thickness were observed, suggesting a more hormonally synchronized and receptive endometrial environment [19,20]. This finding supports the hypothesis that an optimal E2/P4 balance is essential for establishing a timely implantation window, which is critical for IVF success.

Anti-Müllerian hormone (AMH) values also demonstrated significant predictive potential in our study. AMH was positively correlated with E2 levels, the number of oocytes retrieved, endometrial thickness, and pregnancy outcomes. Patients who achieved pregnancy had significantly higher AMH and E2, lower P4, and lower BMI. These findings reinforce the value of combining hormonal and anthropometric parameters to predict IVF success and guide individualized treatment strategies [21]. ROC curve analysis revealed that a P4 threshold of 1.99 ng/mL represents a clinically relevant cutoff with high sensitivity and specificity. Our results support the consideration of a “freeze-all” strategy in cycles with elevated P4, allowing embryo transfer in a subsequent hormonally optimized cycle. Additionally, we observed that term delivery rates were independent of the hormonal parameters analyzed, suggesting that P4 and E2 primarily affect implantation rather than pregnancy maintenance—consistent with similar studies [22,23]. Multivariate regression analysis in our study identified the number of fertilized oocytes and positive pregnancy outcome as the strongest independent predictors of low P4 levels. This suggests that P4 elevation is not an isolated phenomenon but reflects a broader endocrine profile involving ovarian response, endometrial development, and blastocyst competence. In this context, a combined assessment of P4, E2, and ultrasound markers such as endometrial thickness may support the development of personalized embryo transfer strategies and the decision to apply a “freeze-all” approach, as proposed by other research groups [24,25,26]. Although endometrial thickness was not an independent predictor of pregnancy, the significantly higher prevalence of thin endometrium (<7 mm) in patients with elevated P4 (*p* < 0.007) suggests that P4 may influence both morphological and functional endometrial characteristics [27]. Future studies incorporating additional parameters such as endometrial volume, vascularization, and molecular markers of receptivity (e.g., integrins, LIF) may clarify whether the impact of P4 is direct or mediated through E2 and other factors [28].

As in our previous study, patients who achieved pregnancy had lower BMI values, which is in line with the findings of previous meta-analyses linking BMI >25 kg/m^2^ to reduced clinical pregnancy and live birth rates. Non-smoking status was also more prevalent among women with favorable outcomes, supporting the notion that smoking may negatively affect hormonal balance and endometrial receptivity [10,29,30]. Furthermore, the choice of insemination technique (IVF vs. ICSI) was not significantly associated with pregnancy outcome, confirming previous findings that fertilization method is not a key determinant of success when other parameters are optimal [31,32].

These findings underscore the importance of combining hormonal, anthropometric, and clinical factors when evaluating candidates for ARTs, supporting the need for personalized treatment strategies.

As a non-randomized, single-center design without blinding, the findings may be subject to selection bias and limited external validity. Also, its retrospective design, relatively small sample size, and the wide age range of patients (20–40, only one has 44 years) have contributed to variability in ovarian response and IVF outcomes. Additionally, subgroup analysis based on stimulation protocol (agonist vs. antagonist) was not performed, which may have influenced hormonal values. Future studies should stratify hormonal dynamics according to stimulation type, as these protocols may yield differing endocrine profiles. Embryo quality assessment was also not included, which could have provided further insight into the role of P4 in early embryonic development.

## 5. Conclusions

Conversely, higher estradiol concentrations on the day of hCG trigger correlate positively with oocyte yield, fertilization, and clinical pregnancy, but are not independent predictors of implantation. An optimal hormonal environment—characterized by P4 < 2 ng/mL, elevated E2, higher AMH, and endometrial thickness > 7 mm—appears favorable for IVF success. These findings emphasize the importance of comprehensive hormonal profiling and endometrial assessment in guiding embryo transfer timing and tailoring individual stimulation protocols. Implementing a “freeze-all” strategy may be beneficial in cases of premature progesterone rise to preserve implantation potential and optimize clinical outcomes.

## Figures and Tables

**Table 1:** Descriptive age, BMI, and hormonal status, as well as the obtained oocytes and returned embryos.

	Number	Mean	SD	Min.	Max.	Median
**Female age (years)**	128	34.54	4.06	21.00	44.00	35.00
**BMI (kg/m^2^)**	128	22.39	3.04	16.50	34.50	21.60
**Endometrium (mm)**	128	9.36	1.84	6.00	14.00	9.00
**FSH (mIU/mL)**	128	6.90	2.54	0.95	14.80	6.50
**LH (mIU/mL)**	128	5.47	3.16	0.40	25.20	4.80
**E2 (pg/mL)**	128	46.57	22.30	0.60	130.00	40.00
**P4 (pg/mL)**	128	1.59	1.57	0.20	12.50	1.00
**AMH (ng/mL)**	128	2.72	2.70	0.10	14.30	1.87
**E2 (pg/mL) on hCG day**	128	2330.21	1615.69	264.00	7464.00	1894.00
**P4 (ng/mL) on OR day**	128	2.28	1.06	0.45	7.23	2.12
**Number of oocytes**	128	8.93	7.06	1.00	33.00	7.00
**Fertilization rate**	128	4.86	4.70	0.00	32.00	3.00

**Table 2:** Baseline characteristics and procedure outcome depending on P4 level on the day of OR.

		Total		P4-Low (<2 ng/mL)		P4-High (≥2 ng/mL)		Between Groups *p*
		Number	%	Number	%	Number	%	
**BMI (kg/m^2^)**	<25	105	82.03%	49	84.48%	56	80.00%	0.511
	≥25	23	17.97%	9	15.52%	14	20.00%	
**Endometrium thickness**	≤7	12	9.38%	1	1.72%	11	15.71%	**0.007**
	>7	116	90.63%	57	98.28%	59	84.29%	
**Smoking**	No	97	75.78%	45	77.59%	52	74.29%	0.664
	Yes	31	24.22%	13	22.41%	18	25.71%	
**Cause of** **infertility**	Unexplained	35	27.34%	16	27.59%	19	27.14%	0.900
	Ovarian factor	28	21.88%	12	20.69%	16	22.86%	
	Tubal factor	22	17.19%	12	20.69%	10	14.29%	
	Male factor	34	26.56%	14	24.14%	20	28.57%	
	Ovarian and male fac.	9	7.03%	4	6.90%	5	7.14%	
**Stimulation** **Protocol**	Antagonist/short	112	87.50%	49	84.48%	63	90.00%	0.347
	Agonist/Long	16	12.50%	9	15.52%	7	10.00%	
**Number of** **Oocytes recieved**	1–3	33	25.78%	13	22.41%	20	28.57%	0.682
	4–8	42	32.81%	19	32.76%	23	32.86%	
	≥9	53	41.41%	26	44.83%	27	38.57%	
**Mature** **oocytes No**	1–3 MII	35	27.34%	15	25.86%	20	28.57%	0.373
	4–8 MII	46	35.94%	18	31.03%	28	40.00%	
	≥9 MII	47	36.72%	25	43.10%	22	31.43%	
**Insemination method**	ICSI	39	30.47%	13	22.41%	26	37.14%	0.188
	IVF	50	39.06%	26	44.83%	24	34.29%	
	IVF/ICSI	39	30.47%	19	32.76%	20	28.57%	
**Embryo** **quality**	None	4	30.47%	1	1.72%	3	4.29%	0.153
	A + B	111	3.13%	54	93.10%	57	81.43%	
	C + D	13	10.16%	3	5.17%	10	14.29%	
**Outcome**	Non pregnant	61	47.66%	12	20.69%	49	70.00%	**0.000**
	Pregnant	67	52.34%	46	79.31%	21	30.00%	
**Total**		128	100.00%	58	45.31%	70	54.69%	

**Table 3:** Descriptive parameters, hormonal status, and procedure outcomes by P4 levels.

P4 on Day of OR		*N*	Mean	Std. Deviation	95% CI		Minimum	Maximum	Sign.
					Lower	Upper			
**Female age (years)**	<2	58	34.05	4.41	32.89	35.21	21.00	44.00	**0.218**
	≥2	70	34.94	3.74	34.05	35.83	24.00	40.00	
**BMI (kg/m^2^)**	<2	58	22.19	2.82	21.45	22.93	16.50	29.40	**0.494**
	≥2	70	22.56	3.22	21.79	23.33	18.20	34.50	
**Endometrium (mm)**	<2	58	9.58	1.59	9.16	10.00	7.00	14.00	**0.216**
	≥2	70	9.17	2.01	8.69	9.65	6.00	14.00	
**Infertility duration (years)**	<2	58	5.02	2.56	4.34	5.69	2.00	13.00	0.941
	≥2	70	5.05	2.46	4.46	5.64	1.50	12.00	
**FSH (mIU/mL)**	<2	58	6.77	2.42	6.13	7.41	2.60	14.20	**0.613**
	≥2	70	7.00	2.64	6.37	7.63	0.95	14.80	
**LH (mIU/mL)**	<2	58	5.90	3.95	4.86	6.94	0.40	25.20	0.197
	≥2	70	5.11	2.28	4.56	5.65	0.40	18.00	
**E2 (pg/mL)**	<2	58	43.27	19.25	38.21	48.33	1.00	100.00	0.310
	≥2	70	49.30	24.34	43.50	55.10	0.60	130.00	
**P4 (ng/mL)**	<2	58	1.62	1.86	1.13	2.11	0.28	12.50	0.859
	≥2	70	1.56	1.30	1.24	1.87	0.20	6.40	
**AMH (ng/mL)**	<2	58	3.17	3.22	2.32	4.01	0.10	14.30	0.245
	≥2	70	2.35	2.14	1.84	2.86	0.10	11.00	
**E2 (pg/mL) on hCG day**	<2	58	2725.64	1870.24	2233.88	3217.39	264.00	7464.00	**0.031**
	≥2	70	2002.57	1294.19	1693.98	2311.16	280.00	5420.00	
**P4 (ng/mL) on OR day**	<2	58	1.48	0.41	1.37	1.59	0.45	1.99	**0.000**
	≥2	70	2.94	0.98	2.71	3.18	2.00	7.23	
**Number of oocytes**	<2	58	9.98	7.99	7.88	12.08	1.00	33.00	0.204
	≥2	70	8.06	6.11	6.60	9.51	1.00	27.00	
**M II number**	<2	58	2.17	0.82	1.96	2.39	1.00	3.00	0.292
	≥2	70	2.03	0.78	1.84	2.21	1.00	3.00	
**Fertilization rate**	<2	58	5.74	5.35	4.34	7.15	0.00	32.00	**0.010**
	≥2	70	4.13	3.97	3.18	5.08	1.00	19.00	

**Table 4:** Correlations between examined patients’ characteristics and P4 on the day of OR, E2 on the day of hCG, mature oocyte number, endometrium thickness, and outcome of IVF.

		P4 (ng/mL) on OR Day >2<	E2 (pg/mL) on hCG Day	Mature Oocytes No	Endometrium Thickness	Endometrium >7<	Pregnancy
**Female age (years)**	R	0.111	−0.348	−0.232	−0.294	−0.102	−0.074
	P	0.211	0.000	0.008	0.001	0.250	0.408
**BMI (kg/m^2^)**	R	0.018	−0.072	−0.010	−0.122	−0.130	−0.172
	P	0.838	0.418	0.907	0.170	0.145	0.052
**Endometrium (mm)**	R	−0.118	0.223	0.218	1.000	0.495	0.089
	P	0.186	0.011	0.013		0.000	0.317
**Infertility duration (years)**	R	0.030	0.170	0.131	−0.025	0.061	0.121
	P	0.739	0.055	0.140	0.778	0.494	0.175
**Smoking status**	R	0.038	0.062	0.117	−0.005	0.057	0.028
	P	0.667	0.489	0.190	0.958	0.525	0.752
**FSH (mIU/mL)**	R	0.048	−0.507	−0.397	−0.122	−0.072	−0.095
	P	0.589	0.000	0.000	0.169	0.420	0.285
**LH (mIU/mL)**	R	−0.115	−0.010	0.069	0.027	−0.001	0.075
	P	0.198	0.906	0.440	0.764	0.994	0.398
**E2 (pg/mL)**	R	0.090	0.125	0.039	−0.049	0.055	0.056
	P	0.312	0.161	0.659	0.580	0.536	0.529
**P4 (ng/mL)**	R	0.016	0.116	0.148	−0.094	−0.075	−0.038
	P	0.860	0.191	0.095	0.292	0.399	0.674
**AMH (ng/mL)**	R	−0.103	0.624	0.594	0.201	0.109	0.170
	P	0.246	0.000	0.000	0.023	0.220	0.055
**E2 (pg/mL) on hCG day**	R	−0.192	1.000	0.774	0.223	0.173	0.202
	P	0.030		0.000	0.011	0.050	0.022
**P4 (ng/mL) on OR day**	R	0.862	−0.071	0.004	−0.093	−0.208	−0.447
	P	0.000	0.424	0.964	0.298	0.019	0.000
**P4 (ng/mL) on OR day**	R	1.000	−0.192	−0.094	−0.118	−0.239	−0.491
	P		0.030	0.294	0.186	0.007	0.000

**Table 5:** Correlations between examined IVF cycle characteristics and P4 on the day of OR, E2 on the day of hCG, mature oocyte number, endometrium thickness, and outcome of IVF.

		P4 (ng/mL) on OR Day >2<	E2 (pg/mL) on hCG Day	Mature Oocytes No	Endometrium mm	Endometrium >7<	Pregnancy
**Cause of infertility**	R	0.015	0.069	0.074	−0.054	−0.012	−0.035
	P	0.868	0.437	0.408	0.547	0.897	0.698
**Stimulation protocol**	R	−0.083	0.266	0.196	0.159	0.041	0.124
	P	0.351	0.002	0.027	0.074	0.650	0.163
**Number of oocytes**	R	−0.113	0.875	0.896	0.239	0.150	0.156
	P	0.205	0.000	0.000	0.007	0.091	0.078
**Mature oocytes No**	R	−0.094	0.774	1.000	0.218	0.135	0.137
	p	0.294	0.000		0.013	0.127	0.123
**Insemination method**	R	−0.121	0.312	0.220	0.059	0.000	0.080
	p	0.175	0.000	0.012	0.506	1.000	0.368
**Fertilization rate**	R	−0.228	0.626	0.646	0.132	0.127	0.264
	p	0.010	0.000	0.000	0.138	0.152	0.003

**Table 6:** Patient characteristics and average hormonal level and outcome of IVF procedure.

		*N*	Mean	Std. Deviation	Minimum	Maximum	Sign.
**Female age (years)**	Non pregnant	61	34.79	3.97	24.00	40.00	**0.512**
	Pregnant	67	34.31	4.16	21.00	44.00	
	Total	128	34.54	4.06	21.00	44.00	
**BMI (kg/m^2^)**	Non pregnant	61	23.06	3.38	18.70	34.50	**0.016**
	Pregnant	67	21.78	2.56	16.50	29.40	
	Total	128	22.39	3.04	16.50	34.50	
**Endometrium (mm)**	Non pregnant	61	9.17	1.90	6.00	13.00	**0.285**
	Pregnant	67	9.52	1.78	6.50	14.00	
	Total	128	9.36	1.84	6.00	14.00	
**FSH** **(** **mIU/mL)**	Non pregnant	61	7.19	2.94	0.95	14.80	**0.205**
	Pregnant	67	6.62	2.09	3.10	11.40	
	Total	128	6.90	2.54	0.95	14.80	
**LH** **(** **mIU/mL)**	Non pregnant	61	4.98	1.71	0.40	9.90	0.396
	Pregnant	67	5.91	4.02	0.40	25.20	
	Total	128	5.47	3.16	0.40	25.20	
**E2 (pg/mL)**	Non pregnant	61	45.92	23.49	0.60	130.00	0.527
	Pregnant	67	47.16	21.32	1.00	102.00	
	Total	128	46.57	22.30	0.60	130.00	
**P4 (ng/mL)**	Non pregnant	61	1.61	1.38	0.20	6.40	0.672
	Pregnant	67	1.57	1.74	0.28	12.50	
	Total	128	1.59	1.57	0.20	12.50	
**AMH (ng/mL)**	Non pregnant	61	2.25	2.20	0.10	10.70	**0.05**
	Pregnant	67	3.15	3.05	0.10	14.30	
	Total	128	2.72	2.70	0.10	14.30	
**E2 (pg/mL) on hCG day**	Non pregnant	61	2008.95	1471.77	264.00	6540.00	**0.023**
	Pregnant	67	2622.70	1694.61	529.00	7464.00	
	Total	128	2330.21	1615.69	264.00	7464.00	
**P4 (ng/mL) on OR day**	Non pregnant	61	2.64	0.89	1.15	5.35	**0.000**
	Pregnant	67	1.95	1.11	0.45	7.23	
	Total	128	2.28	1.06	0.45	7.23	
**Number of oocytes**	Non pregnant	61	7.66	5.86	1.00	22.00	0.05
	Pregnant	67	10.09	7.87	1.00	33.00	
	Total	128	8.93	7.06	1.00	33.00	
**Fertilization rate**	Non pregnant	61	3.82	3.58	0.00	14.00	**0.003**
	Pregnant	67	5.81	5.37	1.00	32.00	
	Total	128	4.86	4.70	0.00	32.00	
**P4 (ng/mL) on OR day**	Non pregnant	61	1.80	0.40	1.00	2.00	**0.000**
	Pregnant	67	1.31	0.47	1.00	2.00	
	Total	128	1.55	0.50	1.00	2.00	

**Table 7:** Univariant and multivariant regression analysis P4 level < 2 on the day of OR.

Univariate LRA					Multivariate LRA			
	Exp(B)	Sig.	95% C.I. for EXP(B)		Exp(B)	Sig.	95% C.I. for EXP(B)	
			Lower	Upper			Lower	Upper
**Age**	1.056	0.218	0.968	1.152				
**BMI (kg/m^2^)**	1.361	0.512	0.542	3.419				
**Endometrium**	0.094	**0.026**	0.012	0.753	0.304	0.296	0.032	2.842
**FSH**	1.037	0.610	0.902	1.191				
**AMH**	0.891	**0.095**	0.777	1.020	0.950	0.584	0.791	1.141
**E2**	0.460	**0.035**	0.224	0.947	0.801	0.652	0.305	2.102
**Stimulation protocol**	0.605	0.351	0.210	1.739				
**Number of oocytes**	0.825	0.386	0.534	1.275				
**MII oocytes No**	0.795	0.309	0.512	1.237				
**Fertilization rate**	0.924	**0.063**	0.850	1.004	1.029	0.610	0.923	1.146
**Outcome/pregnancy**	0.112	**0.000**	0.049	0.253	0.164	**0.000**	0.068	0.395

## Data Availability

Data are contained within the article.

## Abbreviations

The following abbreviations are used in this manuscript:

ART	Assisted Reproductive Technology
IVF	In Vitro Fertilization
ICSI	Intracytoplasmic Sperm Injection
E2	Estradiol
P4	Progesterone
FSH	Follicle-Stimulating Hormone
LH	Luteinizing Hormone
AMH	Anti-Müllerian Hormone
hCG	Human Chorionic Gonadotropin
OR	Oocyte Retrieval
MII	Metaphase II
BMI	Body Mass Index
ROC	Receiver Operating Characteristic
SPSS	Statistical Package for the Social Sciences
GnRH	Gonadotropin-Releasing Hormone
